# Ethnobotany for food security and ecological transition: wild food plant gathering and consumption among four cultural groups in Kurram District, NW Pakistan

**DOI:** 10.1186/s13002-023-00607-2

**Published:** 2023-09-01

**Authors:** Sayed Taufiq Hussain, Sayed Muhammad, Sheharyar Khan, Wahid Hussain, Andrea Pieroni

**Affiliations:** 1 Department of Botany, GPGC Parachinar, Parachinar, 26300 Kurram District, KP Pakistan; 2 Department of Botany, University of Peshawar, Peshawar, KP Pakistan; 3 University of Gastronomic Sciences, Piazza Vittorio Emanuele II 9, 12042 Pollenzo, Italy; 4 Department of Medical Analysis, Tishk International University, Erbil, 44001 Iraq

**Keywords:** Ethnobotany, Wild food plants, Kurram, Pakistan, Food security

## Abstract

**Background:**

In traditional food systems, especially those of rural populations around the world, wild food plants remain crucial. These resources need to be urgently documented to lay the foundations for sustainable livelihoods and food security.

**Methods:**

In the present field study, we gathered information about wild food plants and mushrooms consumed by four ethnic groups (Turis, Khushis, Hazaras, and Christians) living in Kurram District, NW Pakistan, by conducting semi-structured interviews and holding group discussions.

**Results:**

A total of 57 wild edible plants and mushrooms were reported, with the documented taxa belonging to 50 genera and 34 families. Turis reported the highest number of wild food plants (41), followed by Hazaras (37), Khushis (35), and then Christians, who reported only 11 plants. The most dominant families were Rosaceae, followed by Polygonaceae, Brassicaceae, Fabaceae, Lamiaceae, Moraceae, and Plantaginaceae. The comparative analysis we conducted with the pre-existing Pakistani ethnobotanical studies revealed that 23 wild edible plants have not been previously reported as food items in the area under study, which included *Fragaria nubicola*, *Lepidium draba*, *Pinus wallichiana*, *Podophyllum emodi*, *Prunus jacquemontii*, *Sambucus nigra*, *Sideroxylon mascatense*, and *Thymus linearis*. Four wild edible mushrooms are also reported for the area for the first time: *Calvatia gigantea*, *Morchella esculenta*, *Pisolithus albus*, and *Tulostoma squamosum*. The cross-cultural analysis of wild edible plants and their uses revealed remarkable similarity between Khushis and Hazaras. The overlapping pattern of wild edible plant use among these two groups, as well as Turis, confirms the existence of cross-cultural interactions among these communities, which have shared the same environmental and socio-cultural space for several decades. Food heritage and some unique dishes are linked to wild edible plants in the area, such as *Zamda*, prepared by Turis, and *Saba*, famous among Khushis and Hazaras.

**Conclusion:**

This study suggests that some wild edible plants could be cultivated to protect a few threatened species from overexploitation, while the overall wild food plant heritage should be promoted and revitalized; for example, within educational platforms aimed at improving the wellbeing of local communities and the global ecological transition we must deal with.

## Introduction

Wild edible plants are those plant species, which are not cultivated by humans on farms but rather are collected from the natural habitats in which they are commonly found. The appeal of wild edible plants is considerable because these plants can be used during their active season as well as stored and used in other seasons, such as autumn and winter, when there is no plant growth [1]. Since ancient times, wild edible plants have played a crucial role in shaping human diets. People living in remote areas still use wild botanicals and mushrooms as a source of basic dietary supplements [2]. Functional foods, or foods that can provide not only essential nutritional and energetic needs but also an additional physiological advantage, have attracted increasing attention in recent years. Typically, a food’s functionality depends on some of its constituents, and consumers increasingly choose natural ingredients that are derived from plants. For example, beverages and fermented foods [3] that are preserved locally and also produced from wild edible plants are consumed throughout the world. Ethnobotanical studies in Europe (e.g., Poland, Spain, Portugal, Italy, Bosnia-Herzegovina, France, and some Nordic countries) have provided an impressive overview of (still) existing ethnobotanical knowledge and practices concerning the use of wild edible plants. Many ethnobotanical studies, however, have shown a sudden or gradual decrease in traditional knowledge practices linked to wild foods all over the world [4, 5]. Several field studies that have been conducted during the past two decades in various mountainous regions have revealed that those areas often represent reservoirs of disappearing local plants for food security. Apart from industrialization and globalization, other factors have also detrimentally affected this heritage; the homogenizing effect of centralization in former Soviet territories has, for example, negatively influenced local knowledge linked to plants [6]. The collection and consumption of wild edible plants represent cultural practices which are still followed in many areas of the world and play an important role in food security [7, 8].

Throughout history, wild edible plants have played a crucial role in the human diet. Today, due to the development and advancement of modern agriculture, urbanization, and globalization, human populations are now becoming more distant from their environment. The loss of agricultural practices and wild edible plants have become risks to food security [9]. Recipes based on local plants differ from village to village, as the same species can be cooked in various ways. Some species are perhaps cooked by locals in the same way as in ancient times [10]. People living in developing countries are experiencing difficulty in satisfying their daily needs, thus facing deficiency in one or more micronutrients. In addition, people living in the rural areas of developing countries depend on wild resources, such as wild edible plants and mushrooms, as a food source [11]. Biological and ethnobotanical diversity has given rise to rich bio-cultural heritage as well as ethnobotanical knowledge. However, the development of modern agricultural practices and transport around the globe have created a great variety of cultivated wild edible plants, which can now be easily found in markets [12]. There is no doubt that wild edible plants have been important supplements to a balanced diet, but consumption has been decreasing throughout the world and is currently showing its lowest recorded figures. A decrease in the consumption of wild edible plants results in an unhealthy and unbalanced diet, and this has been linked to various diseases and health conditions, as well as increased mortality rates, which may affect a country’s economy. In every country and region, poverty is a major contributor to food scarcity. Hunger and malnutrition occur as a result of the scarcity of food. In short, in order to maintain a healthy diet, it needs to be balanced [13].

Modern techniques have been used to demonstrate the nutritional potential of wild edible plants [14] and ethnobotanical research is beneficial in the development and discovery of new foods and drugs [15]. Plants play an important role in the life of humans because many people still depend on wild edible plants for fodder, food, medicine, and cosmetics. Throughout the world, but especially in rural areas, wild edible plants play a vital role in curing different diseases as well as overcoming malnutrition. In developing countries, people still use approximately 80% of the wild edible plants available to them to help treat diseases [16]; the number of identified wild edible plants species has been estimated to be 350,000, but around 80,000 are considered safe for human use [17].

Kurram District is one of the most economically and socially disadvantaged areas of Pakistan; it is divided into three main regions: Upper, Central, and Lower Kurram. Researchers contributing to the flora of Pakistan have deposited several specimens from Upper and Lower Kurram in the KEW Herbarium at Peshawar University and Karachi University Herbarium. Central Kurram has not yet been explored floristically, but a few ethnobotanically studies have been conducted [18, 19] in Kurram District. In the ethnobotanical literature of Pakistan, a few studies have been published on the medical ethnobotany of Kurram, yet little fieldwork has been conducted on the wild edible plants used among the different ethnic and religious groups of the region. Therefore, in order to provide local communities the basic tools needed for improving their wellbeing and food security, the primary objectives of the study were to: (a) document the wild food plants traditionally gathered among four ethnic and religious groups living in Kurram District, NW Pakistan; (b) cross-culturally compare the recorded local knowledge among the four considered groups and with the ethnobotanical literature of Pakistan, in order to identify commonalities and differences and possible novel plant reports; and (c) discuss the threats to and potential of this body of applied botanical knowledge and practices.

## Material and methods

### The study area

**Fig. 1:**
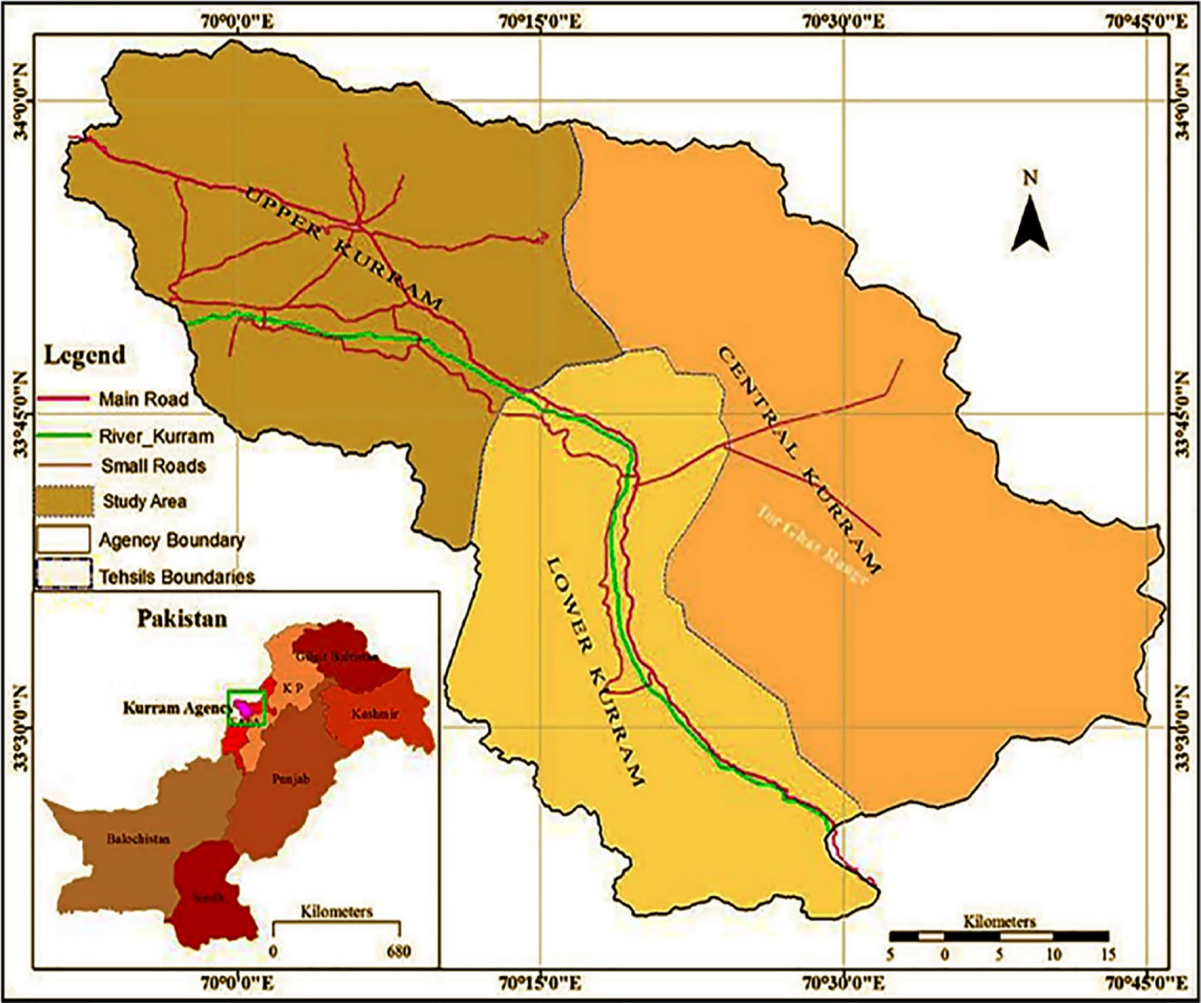
Map of the study area

The tribal agency known as Kurram is a newly formed district of Khyber Pakhtunkhwa, Pakistan. Kurram District is located between 33°20′ north latitude and 69°50′ and 70°50′ east longitude (Fig. 1). The Kurram River flows through the valley, from which the district obtained its name; the name Kurram is mentioned in “rag Vide” and it may be derived from the word *Karma*, although it could have been derived also from the word *Kirram*, which means silk, as people living in the valley once kept silkworms for their livelihood. Kurram District is 115 km long at its greatest extent and covers a total area of 3,380 square kilometers. The area has four distinct seasons; in winter snowfalls are common and the temperature may fall as low as − 10 °C, while in summer the temperature may reach as high as 35 °C. The vegetation of the area consists of dry temperate coniferous forest, alpine forest, and semi-evergreen forest. Dwarf palm, mulberry, poplar, willow, pine, cedar, and oak trees are commonly found in the local landscape.

**Fig. 2:**
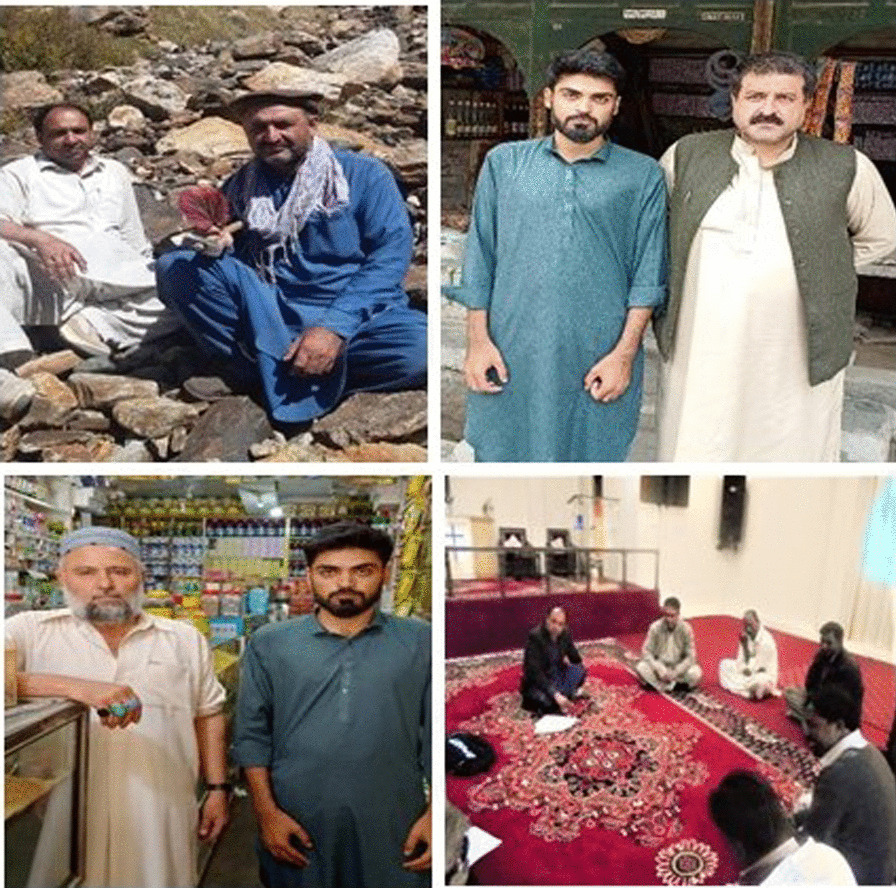
Interviewing local elderly participants during the field study: Turis, Khushis, Hazaras, and Christians

The Kurram Valley was one of the most used routes into India during Roman times [20–22]. The present-day boundary of the Koh-e-Sufaid range appears to be the same as in ancient Svethpatha. It is assumed that the green uplands of the Kurram Valley have represented an ideal arena for humans for millennia, especially for early Hindu and Aryan inhabitants (Fig. 2).

Four ethnic and religious communities were considered in this applied botanical study: Turis, who represent a large majority of the Kurram population; and three tiny minorities: Hazaras, Khushis, and Christians. The following paragraphs provide a brief historical overview of these communities [20–22].

### The studied communities

#### Turis

Turis represent a very distinct tribe among Pashto/Pathan speaking peoples. The Turi tribe may have originated somewhere in western Asia and moved to Kurram around the sixteenth century [20–22]. According to local elders, the tomb of the great-grandfather of the Turi tribe is located in Afghanistan. In the past, Turis were very powerful in the region, and today members of this group represent the ethnic majority of Kurram and can be found everywhere throughout the valley. They are Sunni Muslim.

#### Hazaras

Hazaras represent a very small Shia Muslim minority group in Kurram District, mostly concentrated in Parachinar. They originally came from Hazarajat, a large mountainous region of central Afghanistan, and migrated to Kurram District after the Taliban regime was established in the 1990s. While they previously represented about two thirds of the total population of Afghanistan, over a period of time the majority of Hazaras were either killed (genocide) or forced to flee to other destinations and at present they comprise 20 to 25% of the total population of the country. Among historians there is a consensus that Hazaras are related to the Mongols–Mongol invasions in the area starting in the early thirteenth century. It is said that Genghis Khan and his successors divided their armed forces in groups of Hazar, which means one thousand in Persian; it is therefore believed that the term Hazar later became Hazara and came to represent the identity or nomenclature of the Mongols in this area. One can find several words, names, and terms that appear to be either the same or altered forms of the Mongol language. In addition, several other cultural norms, such as clothing and food preferences, are also similar to that of Mongol culture.

Hazaras speak Hazaragi, a modified dialect of Dari (Persian). The Hazaragi language spoken by the Hazaras of Parachinar, however, has adopted words and phrases from the local Pashtu language, so much so that when two Hazaras speak together, nearby Pashtuns can understand the crux of their discussion. As a result of living among Turi and Bangash tribes of Pashtuns, the Hazara community has developed very strong social contacts with these groups, which have included matrimonial relationships, trade and business partnerships, and strong bonds of friendship along personal and family lines. Most Hazaras speak the local dialect of the Pashtu language but do not have complete command of it. At present, about 120–150 families of Hazaras live in Parachinar. Most of them run shops and businesses, including clothing retailers, grocery and general provision stores, cosmetic shops, and shoe stores. The literacy rate is quite high among the Hazara community, and a number of people are engaged in civil and military services in Pakistan.

#### Khushis

**Fig. 3:**
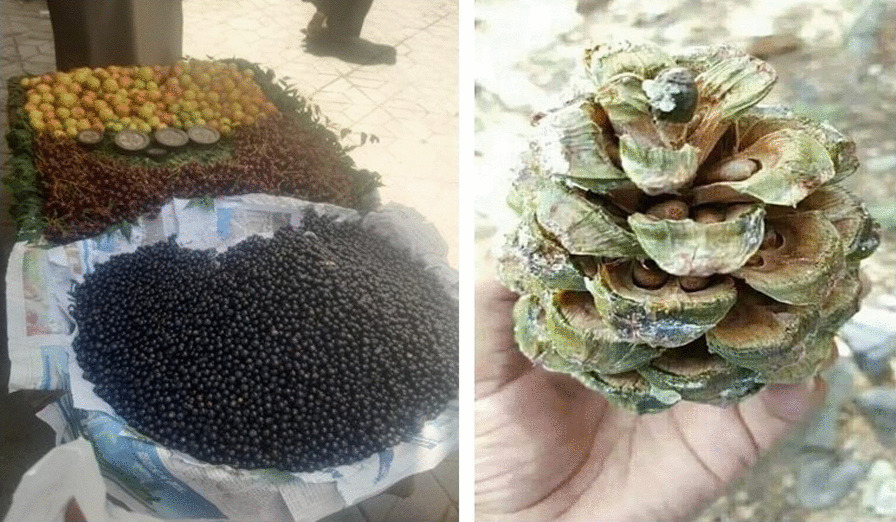
*Sideroxylon mascatense* fruits sold in the market (left) and *Pinus gerardiana* seeds in a mature female cone (right)

Originally belonging to the Logar Province of Afghanistan, most Khushis migrated and settled in and around Parachinar a few decades ago as refugees, after the establishment of the Taliban regime in Afghanistan in the 1990s. The name Khushi refers to the village or valley where they lived in Logar. They are ethnically Tajiks and speak Persian (Dari), and they mainly adhere to Tajik culture in preferences and manners. The majority of Khushis who have settled in Kurram District are Shia, although a few of them profess the Sunni faith. For many elderly people of the area, Khushis are not something new and alien as prior to the Afghan exodus to Pakistan a large number of Khushis used to visit Parachinar for different purposes. There are some families who settled in Parachinar in the late 19th and early twentieth centuries and have lived in the area ever since, where they are mainly involved in trade and related activities. However, their numbers are very small compared to those who crossed over during the Afghan wars. The old Khushis families are well known to everyone in Kurram, and they have established good social contacts and relations with the other Pashtun and non-Pashtun peoples. The common faith of the Khushis, Turis, and Bangash of Kurram have proved to be of great help to many non-Pashtun communities in the otherwise hostile landscape where Pathans normally strictly adhere to their customary laws and very rarely accommodate foreigners within their ranks (Fig. 3).

#### Christians

Christians migrated to Kurram from different parts of Pakistan including Jhelum, Lahore, Karachi, and the Punjab region about one century ago. They settled in Kurram District before Independence Day (14 August 1947), and the community was established by Hindus at that time, who later converted to Christianity. Today, Christians are present in three communities in Parachinar City, as well as the outskirts of the city. They are mainly engaged in urban employment.

### Data collection

This study was conducted in different parts of Kurram District between September 2020 and September 2022. The data were collected from villages as well as cities in Kurram, such as Parachinar in Upper Kurram. The interviews were conducted either individually or in small groups. A total of 120 interviews were conducted among Turis, Khushis, Hazaras, and Christians—30 from each group (Fig. 2). (Table 1). All informants were interviewed in the local language, Pashtu, but some interviews were conducted in Urdu, as Christians are familiar with Urdu and not Pashtu. All of the informants were locals, and the majority of them lived in villages. Because of the cultural constraints of the valley, face-to-face interviews were not possible with women; however, interviews with women were conducted through the men of the different households in their respective groups. The average age of informants was 62 years, with women ranging from 25 to 80 years, and men from 22 to 102 years. The study participants were mainly chosen from among elderly farmers, shepherds, and wild plant collectors and also local markets were visited (Fig. 3). Locals were asked to free list all wild plants and mushrooms they gather or consume within the household. A questionnaire was used for facilitating and guiding more specific questions on each item named during the interviews. The entire field study was conducted following the Ethical Code of the International Society of Ethnobiology [23]. Voucher specimens were identified by the corresponding author according to *Flora of Pakistan*, as in all standard ethnobotanical works recently conducted in the area [18–20, 24–26]. The specimens are stored at the Herbarium of the Department of Botany, GPGC Parachinar. Nomenclature was standardized using the *Word Flora Online* database [27] for plant taxa and the *Index Fungorum* [28] for fungal taxa, while plant family assignments followed the rules of the Angiosperm Phylogeny Website (version 14), as is customary in all contemporary ethnobotanical investigations, including those conducted by our research group [5–7].

**Table 1:** Demographic details of the sample (study participants from the four studied ethnic and religious groups)

Variable	Category	Number of informants	Percentage
Sex	Women	25	21.83%
	Men	95	79.16%
Age	Between 20 and 40 years	27	22.5%
	Between 41 and 60 years	46	83.33%
	Above 60 years	47	39.16%
Education	Illiterate	44	36.67%
	Matriculation	36	30%
	Intermediate	44	36.66%
	Graduate	16	14.16%
Livelihood	Farmer	24	20%
	Shepherd	18	15%
	Plant collector	32	26.66%
	Unspecified (elder)	29	24.16%
	Healer	03	2.5%
	Gardener	06	5%
	Shopkeeper	04	3.33%
	Trader	04	3.33%
Location	Town area	78	65%
	Remote area	42	35%

### Data analysis

The data gathered among the four ethnic and religious groups were compared using the Jaccard similarity index [29]; in ethnobotany, during the past decade, this index has become a useful, although a bit rudimentary, tool for assessing the similarity between two different sets of items.

$${\text{Jaccard}}\;{\text{index}}\; = \;\left( {{\text{the}}\;{\text{number}}\;{\text{in}}\;{\text{both}}\;{\text{sets}}} \right){/}\left( {{\text{the}}\;{\text{number}}\;{\text{in}}\;{\text{either}}\;{\text{set}}} \right)*{1}00.$$

The formula notation is written as:

$$J\left( {X,Y} \right) = \left| {X \cap Y} \right|{/}|X \cup Y|$$

In order to assess possible novelty in the use of wild edible plants, the data were compared with the relevant ethnobotanical literature of Pakistan, specifically focusing on wild foraged plants [14–16, 18, 19, 24, 25, 30–38].

## Results and discussion

### Wild edible plant gathering in the study area

The traditional food system of the four ethnic and religious groups in Kurram is based on seasonal wild food plants, consisting of three main groups of foraged items: wild vegetables and mushrooms, wild seasoning plants, and wild fruits. While most wild plants and mushrooms are consumed fresh, while only a few species are used during the off-season, i.e., winter, in dried form.

**Fig. 4:**
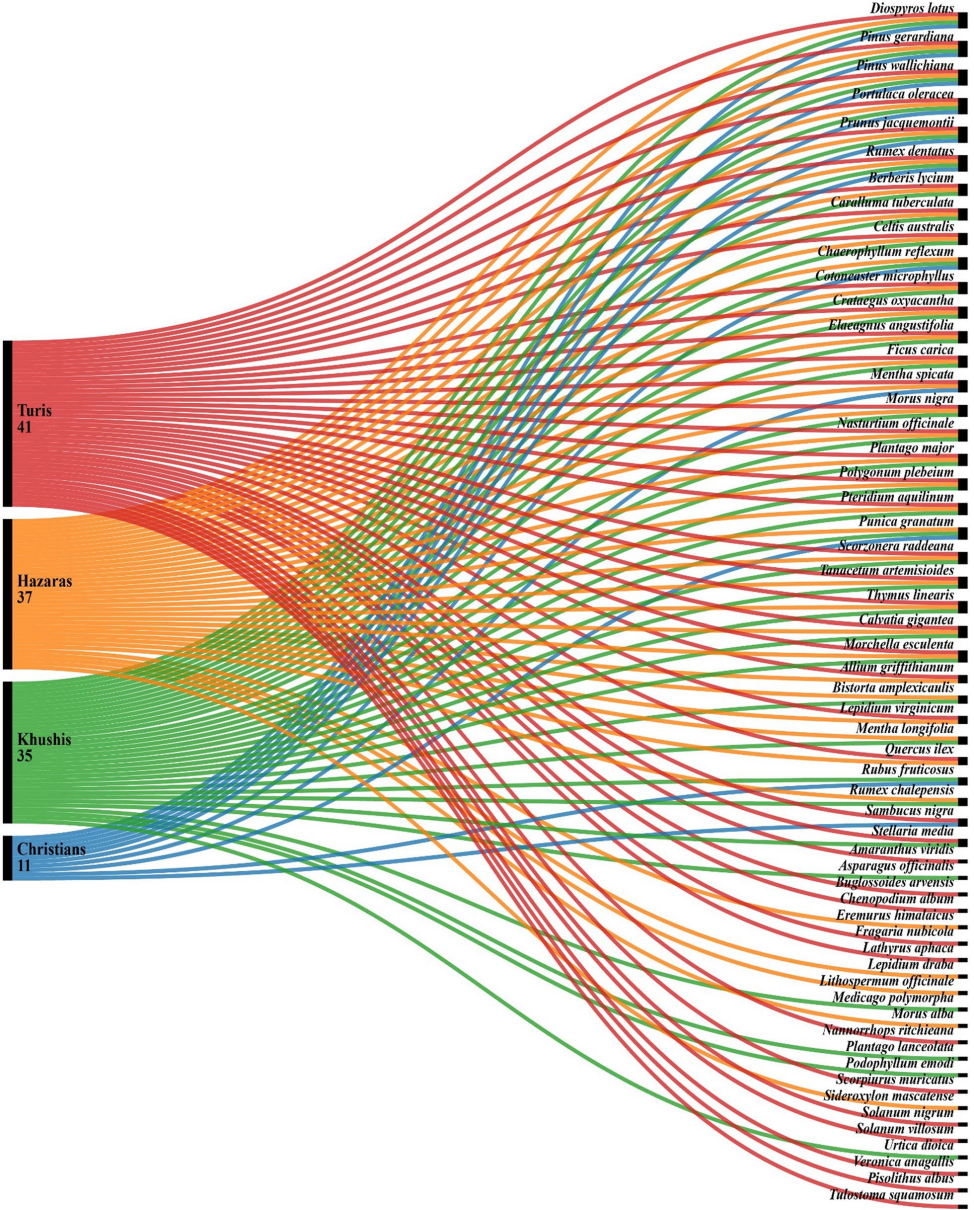
Alluvial diagram illustrating the distribution of plant uses among the studied groups

The ethnobotanical data on wild edible plants and mushrooms, their used parts, and their methods of use are presented in Table 2. A total of 57 wild edible plants from 50 genera and 34 families were recorded. The specimens were collected, preserved, and identified by one of the authors (W.H.) and then deposited in the herbarium of the Department of Botany, GPGC Parachinar, Kurram. The most dominant plant families are Rosaceae (five species), followed by Polygonaceae (four species), Moraceae, Fabaceae, Brassicaceae, Lamiaceae, and Plantaginaceae (three species each). Among the documented plants, 41 were mentioned by Turis, 35 by Khushis, 37 by Hazaras, and 11 by Christians (Fig. 4).

**Table 2:** Foraged wild food plants and mushrooms among the studied groups of Kurram District, NW Pakistan

Botanical Taxon, Family, and Botanical Voucher Code	Local Name	Used Plant Part(s)	Traditional Food Use	Tur	Khu	Haz	Chr	Other Reports in Pakistan
*Allium griffithianum* Boiss., Amaryllidaceae*,* M.T.171.GPGC.PCR	Pizaki	Whole plant	Seasoning	+	−	+	−	[24, 32]
*Amaranthus viridis* L Amaranthaceae*,* M.T.172.GPGC.PCR	Rinzaka	Leaves	Cooked	+	−	−	−	[31–33]
*Asparagus officinalis* L Asparagaceae*,* M.T.173.GPGC.PCR	Lakhtey	Stems	Cooked	−	+	−	−	[24]
*Berberis lycium* Royle Berberidaceae, M.T.174.GPGC.PCR	Toorwokay	Fruit	Raw	+	+	+	−	[15, 20, 34]
*Bistorta amplexicaulis* (D.Don*)* Greene Polygonaceae, M.T.175.GPGC.PCR	Sarka	Leaves	Raw	−	+	+	−	[24, 33]
*Buglossoides arvensis* (L.) I.M. Johnst Boraginaceae*,* M.T.176.GPGC.PCR	Spir saba	Leaves	Cooked	+	−	−	−	[24]
*Caralluma tuberculata* N.E.Br Apocynaceae*,* M.T.177.GPGC.PCR	Pamani	Whole plant	Raw in salads	+	+	+	−	[31–35]
*Celtis australis* L Cannabaceae*,* M.T.178.GPGC.PCR	Toogh	Fruit	Raw	+	+	+	−	[20, 31]
*Chaerophyllum reflexum* Aitch Apiaceae*,* M.T.179.GPGC.PCR	Zanrrkay	Leaves	Cooked	−	+	+	+	[15–19, 24]
*Chenopodium album* L Amaranthaceae, M.T.180.GPGC.PCR	Sormay	Leaves	Cooked	+	−	−	−	[15, 31–34]
*Cotoneaster microphyllus* Wall.ex Lindl Rosaceae*,* M.T.181.GPGC.PCR	Kherawaa	Fruit	Raw	+	+	+	−	[19]
*Crataegus oxyacantha* L Rosaceae*,* M.T.182.GPGC.PCR	Ghowanza	Fruit	Raw	+	+	+	−	
*Diospyros lotus* L Ebenaceae*,* M.T.183.GPGC.PCR	Toor amlook	Fruit	Raw	+	+	+	+	[20, 34, 36]
*Elaeagnus angustifolia* L., Elaeagnaceae*,* M.T.184.GPGC.PCR	Seenzala	Fruit	Raw	+	+	+	−	[18]
*Eremurus himalaicus* Baker, Asphodelaceae/Xanthorrhoeaceae, M.T.185.GPGC.PCR	Hazee	Leaves	Cooked	−	−	+	−	[24]
*Ficus carica* L Moraceae*,* M.T.186.GPGC.PCR	Inzaar	Fruit	Raw and cooked	+	+	+	−	[20, 34, 36]
*Fragaria nubicola* (Lindl. ex Hook.f.) Rosaceae*,* M.T.187.GPGC.PCR	Waraa Manzakhaka	Fruit	Raw	+	−	−	−	[15, 24, 34]
*Lathyrus aphaca* L Fabaceae*,* M.T.188.GPGC.PCR	Marghayo hpay	Leaves	Cooked	+	−	−	−	[24, 31]
*Lepidium draba* L Brassicaceae M.T.189.GPGC.PCR	Bashkay	Leaves	Cooked	−	−	+	−	[24]
*Lepidium virginicum* L Brassicaceae*,* M.T.190.GPGC.PCR	Zangali Teraba	Leaves	Raw in salads	+	−	+	−	[24]
*Lithospermum officinale* L Boraginaceae*,* M.T.191.GPGC.PCR	Noraki Saba	Whorls of growth	Cooked	−	−	+	−	[25]
*Medicago polymorpha* L Fabaceae*,* M.T.192.GPGC.PCR	Kundi	Leaves	Cooked	−	+	−	−	[31, 33]
*Mentha longifolia* (L) L Lamiaceae*,* M.T.193.GPGC.PCR	Villanay	Leaves	Raw in salads	−	+	+	−	[15, 24, 20, 31]
*Mentha spicata* L Lamiaceae*,* M.T.194.GPGC.PCR	Podina	Leaves	Raw in salads	+	−	+	+	[32–34]
*Morus alba* L Moraceae*,* M.T.195.GPGC.PCR	Speen toot	Fruit	Raw in salads	−	−	+	−	[20, 36]
*Morus Nigra* L., Moraceae*,* M.T.196.GPGC.PCR	Toor toot	Fruit	Raw in salads	+	+	+	−	[20, 36]
*Nannorrhops ritchieana* (Griff.) Aitch Arecaceae*,* M.T.197.GPGC.PCR	Pataweey	Fruit	Raw	+	−	−	−	[18, 37]
*Nasturtium officinale* R.Br Brassicaceae*,* W.A.198.GPGC.PCR	Shiree	Young stems and leaves	Raw in salads	+	+	+	−	[24, 31, 33]
*Pinus gerardiana* Wall.ex D.Don Pinaceae*,* M.T.199.GPGC.PCR	Chalghoza	Seeds	Raw	+	+	+	+	[35]
*Pinus wallichiana* A.B Jacks Pinaceae*,* M.T.200.GPGC.PCR	Neenjaraa	Seeds	Raw	+	+	+	+	[20, 34]
*Plantago lanceolata* L Plantaginaceae*,* M.T.201.GPGC.PCR	Poly spor	Leaves	Cooked	−	+	−	−	[15, 24, 20, 34]
*Plantago major* L Plantaginaceae*,* M.T.202.GPGC.PCR	Ghayo Zhabey	Leaves	Cooked	+	+	+	−	[15, 24, 20, 34]
*Podophyllum emodi* wall.ex Hook.f. & Thomson, Berberidaceae*,* M.T.203.GPGC.PCR	Ghara marchaka	Fruit	Raw	−	+	−	−	[34]
*Polygonum plebeium* R.Br., Polygonaceae*,* M.T.204.GPGC.PCR	Bandoki	Stems	Cooked	+	+	+	−	[24, 34]
*Portulaca oleracea* L., Portulaceae*,* M.T.205.GPGC.PCR	Varhori	Leaves and stems	Cooked	+	+	+	+	[24, 34, 35]
*Prunus jacquemontii* Hook.f Rosaceae*,* M.T.206.GPGC.PCR	Arghenja	Fruit	Raw	+	+	+	+	[18]
*Pteridium aquilinum* (L.) Kuhn Dennstaedtiaceae*,* M.T.207.GPGC.PCR	Kawsay	Leaves	Cooked	+	+	+	−	[24]
*Punica granatum* L*.,* Punicaceae, M.T.208.GPGC.PCR	Wangaar	Fruit	Raw	−	+	+	+	[20, 31, 34]
*Quercus ilex* L., Fagaceae*,* M.T.209.GPGC.PCR	Pargee	Seeds	Cooked	+	−	+	−	[20]
*Rubus fruticosus* G.N.Jones., Rosaceae*,* M.T.210.GPGC.PCR	Spengir	Fruit	Raw	−	+	−	+	[18, 20]
*Rumex chalepensis* Mill., Polygonaceae*,* M.T.211.GPGC.PCR	Spin Zamda	Leaves	Cooked	−	+	+	−	[24, 32]
*Rumex dentatus* L., Polygonaceae*,* M.T.212.GPGC.PCR	Zamda	Leaves	Cooked	+	+	+	+	[19, 20]
*Sambucus nigra* L., Adoxaceae*,* M.T.213.GPGC.PCR	Lantus	Fruit	Raw	+	−	−	+	[20]
*Scorpiurus muricatus* L., Fabaceae*,* M.T.214.GPGC.PCR	Spinki saba	Leaves	Cooked	+	−	−	−	[24]
*Scorzonera raddeana* C. Winkl., Asteraceae*,* M.T.215.GPGC.PCR	Mutayer	Leaves	Cooked	+	+	+	−	[24]
*Sideroxylon mascatense* (A.DC.) T.D.Penn Sapotaceae*,* M.T.216.GPGC.PCR	Gorgoray	Fruit	Raw	−	−	+	−	[37]
*Solanum nigrum* L., Solanaceae*,* M.T.217.GPGC.PCR	Toor Parsubay	Fruit and leaves	Raw	+	−	−	−	[18, 24, 34]
*Solanum villosum* Mill., Solanaceae*,* M.T.218.GPGC.PCR	Soor Parsubay	Fruit and leaves	Raw	+	−	−	−	[24]
*Stellaria media* (L.) Vill., Caryophyllaceae*,* M.T.219.GPGC.PCR	Vilaghori/ Badsha saba	Leaves	Cooked	+	+	−	−	[24]
*Tanacetum artemisioides* Sch.Bip. ex Hook.f Asteraceae, M.T.220.GPGC.PCR	Zaveel	Leaves	Raw	+	+	+	−	[20]
*Thymus linearis* Benth Lamiaceae*,* M.T.221.GPGC.PCR	Panni	Leaves and stems	Seasoning	+	+	+	−	[15, 20, 34, 39]
*Urtica dioica* L Urticaceae*,* M.T.222.GPGC.PCR	Sezoonki	Leaves	Cooked	−	+	−	−	[15, 34, 39]
*Veronica anagallis*L Plantaginaceae, M.T.223.GPGC.PCR	Obo Saba	Leaves	Cooked	+	−	−	−	[24]
*Calvatia gigantea* (BatschexPers.) Lloyd, Agaricaceae, M.T.224.GPGC.PCR	Sheeshtee	Fruiting body	Cooked	+	+	+	−	
*Morchella esculenta* Fr., Morchellaceae, M.T.225.GPGC.PCR	Kilkichok	Fruiting body	Cooked	+	+	+	−	
*Pisolithus albus* (priest ex Bougher & K.Syme.) Sclerodermataceae, M.T.226.GPGC.PCR		Fruiting body	Cooked	+	−	−	−	
*Tulostoma squamosum* (J.F.Gmel.) Pres Agaricomycetes, M.T.227.GPGC.PCR		Fruiting body	Cooked	+	−	−	−	

*Tur* Turis, *Khu* Khushis, *Haz* Hazaras, *Chr* Christians; + recorded gathering/food use; − no recorded gathering/food use

In Kurram, the main source of income and food is agriculture. The majority of the population of Kurram depends on cultivation for their livelihood. The major crops include wheat, rice, potato, tomato, and maize. However, wild edible plants also play a key role in the daily life of the people in the Kurram Valley. In Kurram, the Turi tribe has a large population compared to the other ethnic and religious groups because they have lived there since pre-historic times, and they have used wild edible plants for a long time; they also use wild plants for commercial purposes and to make a profit as they sell some of these wild botanicals, such as *M. spicata* and *C. tuberculata*, in the market. The most common wild greens consumed by Turis are *P. major*, *A. griffithianum*, *A. viridis*, *A. officinale*, and *B. amplexicaulis*, whereas Khushis mainly consume *C. reflexum* and *C. microphyllus*, Hazaras *E. himalaicus*, *Lipidium verginicum*, and *L. officinale*, and Christians *M. spicata* and *P. oleracea* [33].

Wild edible fruits play a role in the daily life of the four ethnic and religious groups in Kurram. Of the 57 recorded wild edible plants, 16 were fruits. The wild edible fruits most commonly reported by Turis include *F. carica*, *C. australis*, *D. lotus*, *F. nubicola*, and *S. mascatense* (Fig. 3).

Khushis mainly use *E. angustifolia*, *M. alba* (dried), and *P. gerardiana* (*Chilghoza*), while Hazaras top used wild fruits are those of *P. gerardiana* and *F. carica* (dried) and Christians use *M. alba*, *D. lotus*, *F. carica*, and *M. nigra*. Overall, *P. jacquemontii* is the most common wild edible fruit of Kurram District.

In terms of foraging, and not only using/consuming, the degree of plant collection among the four ethnic and religious groups varies remarkably, depending on such factors as area of residence, occupation, and regular exposure to the natural environment. Turis live in the foothills of Kurram District and also travel to mountain peaks in search of wild edible plants such as *P. emodi*, which is only found at high elevations. Turis represent the primary group that collects wild edible plants and mushrooms, some of which they sell in the market. Khushis showed less affinity toward plant collection, as they mainly buy wild edible plants in the market. Among Hazaras, only two informants were found to be very knowledgeable about wild edible plants. One of them owns a shop in the market, while the other one occasionally collects wild edible plants. Christians have no knowledge about foraging, mainly because live in the city and have government jobs in Kurram District; they are not connected to the natural environment and therefore reported very few taxa; however, some of their priests seem to have a little knowledge about foraged plants [14].

### Gender roles and traditional local heritage cuisine

During the interviews, the informants were asked qualitative information about the collection and utilization of wild edible plants. The respondents explained that men of the Turi tribe do not much care for collecting wild edible plants. They buy wild edible plants in the market, but also collect some edible plants from the mountains at high elevations. The women of the Turi tribe play a prominent role in foraging wild plants, as they are easily accessible on farms, along riverbanks, and in the foothills of mountains. Conversely, women of the Hazara tribe usually do not collect wild edible plants because they are urban inhabitants concerned with domestic affairs; thus, they do not visit wild hillsides. However, despite the fact that most Khushis and Hazaras live in the main city, wild edible plants are collected by Hazara men as they sometimes visit mountainous areas. Christians in the Kurram Valley do not collect wild edible plants, as they are more concerned with their occupations, but rather mostly buy them in markets.

**Fig. 5:**
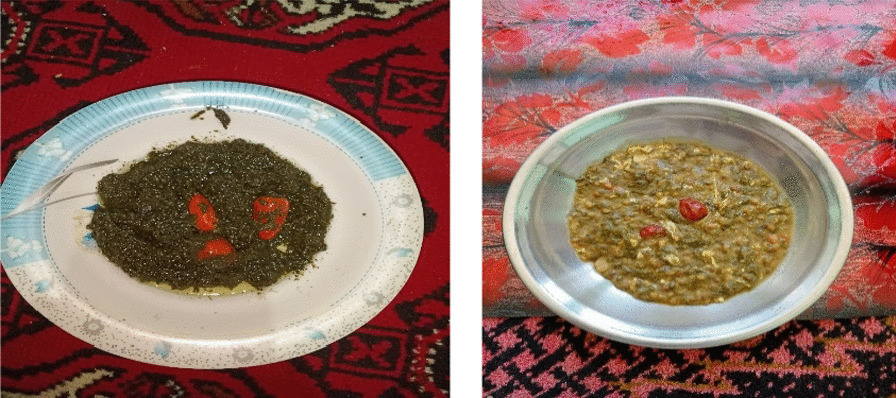
The most common wild food culinary preparations: *Zamda* (prepared with *Rumex dentatus*) (left) and *Saba* (prepared with *Plantago lanceolata*) (right)

One of the most prominent wild vegetable dishes of the Kurram Valley is known as *Zamda* (Fig. 5), cooked by Turi women, which is made from *R. dentatus* (also called *Zamda* and therefore culturally salient) and peppers (*Capsicum annum*). To prepare this dish, *R. dentatus* is boiled in water with peppers for up to half an hour; then, the mixture is fried in oil for up to fifteen minutes [24]. Another popular dish in the Kurram Valley made from wild edible vegetables is known as *Saba*, especially popular among Kushis and Hazaras, which is prepared by mixing *A. viridis*, *A. griffithianum*, and garlic (*Allium sativum*)*.* First, these plants are boiled in water for up to half an hour; then, the mixture is fried in oil with red chilies for up to ten minutes, after which it is ready to eat. Other dishes made by the inhabitants of Kurram District are prepared by combining various wild edible plants; for example, *P. oleracea*, *R. dentatus*, and *S. muricatus* can be made in the same way, but they have a different taste; people also add a fried egg to enhance the flavor of these dishes.

Some other important dishes recorded from the study area include *Marchobaye* (starter), which is made from a mixture of *P. jacquemontii*, *M. spicata*, coriander (*Coriandrum sativum*), chilies, salt, and water, and *Perakaye*, which is a mixture of *Trifolium repens*, eggs, vegetable oil, and wheat flour.

These dishes are prepared all across the valley, especially during the celebration of “Nurooz”, which takes place every year on the 21st of March. “Nurooz” is a Persian word which means “new day” because on this day the grasslands are green and lush and thus available for grazing. The above-mentioned traditional dishes were recorded among families living in the mountainous regions of Kurram, while the consumption of wild plants was considerable for those families living in the most isolated locations of the valleys [35].

Another locally famous preparation is a jam made from *P. jacquemontii*, in which the fruits are first boiled with sugar until the mixture becomes viscous; then, the mixture is cooled in the fridge for up to one hour, after which it is ready to eat. One tasty porridge that is made by locals in Kurram District is known as *Soji halwa*, which is prepared by combining soji flour (semolina), oil, and the nuts of *J. regia.* To make this dish, soji flour is fried with oil for up to 30 min over a medium flame, then some sugar is added for taste, and about 100 g of *J. regia* nuts are added to the flour, which is cooked for up to 20 more minutes. The people of Kurram eat this *halwa* (Pudding) for lunch. Some wild edible plants are eaten raw, such as *D. lotus*, *E. angustifolia*, *F. carica*, *Juglans regia*, *S. mascatense* (Fig. 3), and other species, as documented in Table 2 [18].

**Fig. 6:**
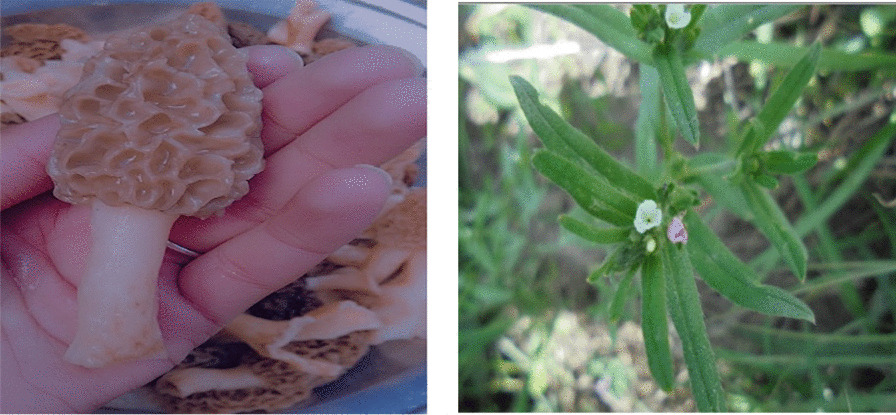
*Morchella esculanta* (left) and *Lithospermum officinale* (right), the most common wild food plants used by Turis, Khushis, and Hazaras

Among the studied ethnic and religious groups, only Turis are involved in collecting wild edible mushrooms. The study recorded four wild edible mushroom taxa among Turis: *C. gigantea*, *M. esculenta*, *P. albus*, and *T. squamosum* (Table 2). The first two of these mushrooms are also used by Hazaras and Khushis. *M. esculenta* is considered highly nutritional and, as a result, it is also very costly when available in the local market (Fig. 6); in terms of preparation, this mushroom is normally fried with tomatoes and salt.

### Cross-cultural analysis

**Fig. 7:**
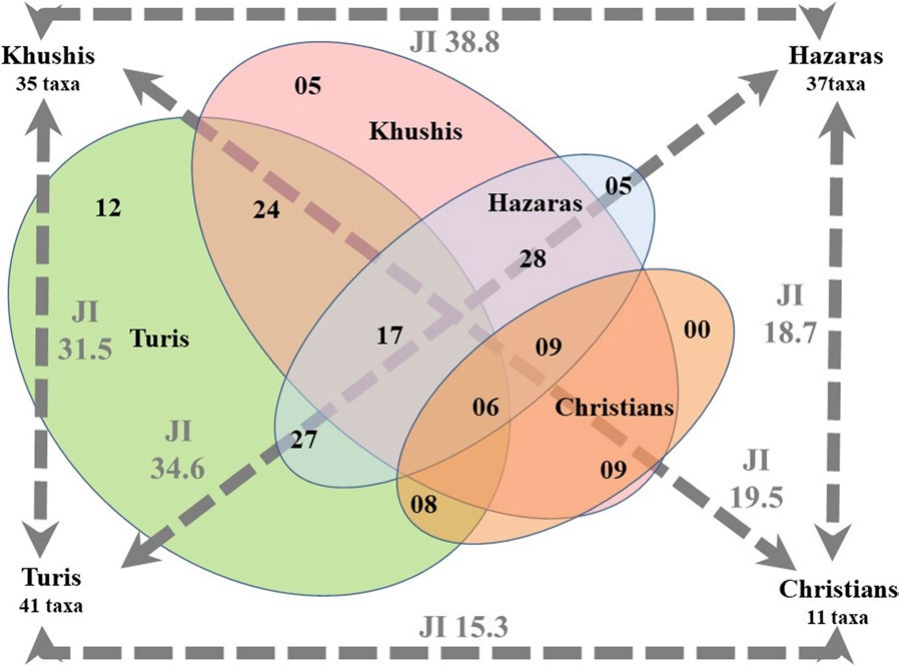
Venn diagram showing the overlapping of wild edible plants among the four studied ethnic and religious groups, as well as their Jaccard indexes (JI)

A comparative assessment of wild edible plant use was carried out among the four ethnic and religious groups of Kurram Valley. The largest number of taxa were reported among Turis, as this group has a large population and is more interested in wild edible plants compared to the other three groups, since Turis mostly live in rural and mountainous regions [24]. As Fig. 7 shows, Turis share 24 wild edible plants with Khushis, 27 plants with Hazaras, and eight plants with Christians. Khushis and Hazaras share 28 wild edible plants, while Khushis and Christians share nine such plants. Likewise, Hazaras and Christians share nine wild edible plants. Turis, Khushis, and Hazaras share 17 wild edible plants, while all four ethnic and religious groups share six plants.

Turis, representing the most widespread ethnic group in the valley, reported the largest number of idiosyncratically used wild edible plants (Fig. 7). These 12 wild edible plants are not shared with the other three ethnic and religious groups. Khushis reported five uncommon wild edible plants, as did Hazaras, while Christians did not mention the use of any uncommon wild edible plants. The largest number of plant taxa was also reported by Turis, while the lowest number was reported by Christians. The greatest similarity among the four ethnic and religious groups was recorded for Hazaras and Khushis. However, the least similarity was found between Turis and Christians, as they also have less similarity with Khushis and Hazaras. It is assumed that one third of the reported plant taxa was used by all ethnic and religious groups of the valley.

Turis have been living in the area for a long time and they have their own culture, norms, and values, which are quite distinct from the other three ethnic and religious groups. Turis have long used their traditional rituals, such as those related to marriages, other ceremonies, and plant usage; for example, Turis use the whole plant of *M. longifolia*, while Khushis and Hazaras use only the seeds of this species. The Turi tribe shows the largest number of differences with Christians, followed by Khushis and Hazaras.

Khushis and Hazaras both migrated from Afghanistan, and thus they show a large number of similarities, as reflected in the Jaccard index value (38.8%). In addition to a shared migration, the two groups share the same language and culture. The similarity in wild edible plant use is also due to the high rate of intermarriages between Khushis and Hazaras, which allows for the transfer of knowledge from one group to the other [24, 34]

In terms of wild plant use, Christians show less similarity to the other three groups, perhaps due to their different religion; the other three groups are all Muslim and thus they do not intermarry with Christians, and this may have limited social interactions within the domestic arena, where local foraging and cooking practices are generally transmitted. Another possible reason for the dissimilarity is that Christians live and work in urban areas and so they are not very connected to the agricultural landscape and natural environment [24]; however, this also applies to Hazaras, some of whom have a remarkable (even if not ubiquitous) knowledge of foraging.

### Data novelty, threats, and possibilities of ecological transition

The comparative analysis we conducted with the pre-existing Pakistani ethnobotanical studies revealed that 23 wild edible plants have not been previously reported as food items in the area under study, including *Fragaria nubicola*, *Lepidium draba*, *Pinus wallichiana*, *Podophyllum emodi*, *Prunus jacquemontii*, *Sambucus nigra*, *Sideroxylon mascatense* and *Thymus linearis*. Four wild edible mushrooms are also reported for the area for the first time: *Calvatia gigantea*, *Morchella esculenta*, *Pisolithus albus*, and *Tulostoma squamosum* (Table 2). The remaining 34 species had previously been recognized as being consumed in other areas of Khyber Pakhtunkhwa and elsewhere in Pakistan.

During our field study, we observed that some wild edible plants are facing serious threats, which may endanger these species in the near future. According to informants, the availability of some wild plants has decreased significantly as a result of anthropogenic pressures, such as unhealthy farming practices. Another important factor is the abandonment of wild edible plants because of the availability of industrial foods in markets; wild edible plants are also decreasing due to institutional negligence.

During this study, some endangered species were also recorded, such as *Caralluma tuberculata*, *Ficus carica*, *Lepidium virginicum*, *Lithospermum officinale*, *Nasturtium officinale*, and *Podophyllum emodi* [33]. The four reported mushroom species are also declining considerably. Moreover, some detrimental behaviors were observed while collecting wild edible plants. Because of the unfamiliarity with agricultural practices, farmers sometimes mistakenly collected plants with roots, even if not needed. This practice may reduce the growth of wild edible plants, such as *P. emodi*, which is mostly found in the foothills of mountains. Thus, wild edible plants growing at high elevations in the mountains may become endangered in the near future if the government does not take serious action to protect them. To overcome these challenges, authorities should take decisive measures and, especially, arrange public awareness programs for the protection of these plants.

Moreover, some taxa as *A. griffithianum*, *L. officinale*, *M. spicata*, and *P. oleracea* are used in large quantities, as locals regularly cook them during the spring. Thus, the over-usage of the first two species (*A. griffithianum* and *L. officinale*) may result in the future decrease of their availability. Additionally, people living in mountainous regions and villages also sell some of these plants in local markets, and this could soon make gathering unsustainable [37].

On the other hand, wild edible plants and this body of applied botanical knowledge and practices provide basic food security in developing countries by helping overcome food deficiencies, as well as by providing nutritional supplements to people living in mountainous regions. Wild edible plants also help overcome food insecurity among poorer families that cannot regularly buy cultivated vegetables from markets. The present study reveals that wild edible plants are very important for some locals, generating income for families, and activating small-scale circular economies, especially for those living in mountainous areas. Therefore, the sustainable gathering of wild edible plants may represent a robust strategy for achieving food security. The main goal of all stakeholders in the study area should be that of reducing food insecurity in areas where most of the people cannot live a comfortable life, and incorporating botany into the local strategy of food procurement may play a vital role, especially if sustainable gathering/foraging practices are properly revitalized, including in schools.

Moreover, food security and ecological transition are closely linked, as the way food is produced and consumed has a major impact on the environment and can either support or hinder our ability to ensure a stable and sustainable food supply for future generations. Specifically, food security refers to the ability of individuals and communities to access sufficient, safe, and nutritious food to meet their dietary needs and preferences. This requires not only an adequate food supply, but also access to education, income, and other resources that support healthy eating habits, such as specific measures aimed at promoting local food and foodways [38].

Sustainable foraging and wild food plant consumption can therefore support both ecological transition and food security. Practices such as small-scale and regenerative agriculture and attached foraging practices, as utilized in the study area for centuries, can help to increase biodiversity and reduce greenhouse gas emissions, while also producing nutritious food for local communities. By prioritizing local and seasonal produce and supporting local small-scale producers and foragers, locals could also create more resilient and equitable food systems that are better able to withstand disturbances and ensure everyone has access to healthy food [14, 24, 38]. In summary, promoting local wild plant foods and therefore improving food security is quintessential for the ecological transition. By shifting toward sustainable foraging practices and supporting equitable and resilient local food systems, local  could be able to create a healthier and more sustainable future for ourselves and the planet.

This study can potentially promote entrepreneurial opportunities within the local communities by exploring and harnessing the economic potential of wild food plant gathering and consumption. This may lead to the development of small businesses and initiatives related to the sustainable harvesting, processing, and marketing of these resources. The findings of this study can contribute to the development of ecotourism in Kurram District and its surrounding regions as well. Highlighting the cultural significance and ecological importance of wild food plant gathering practices can attract vistors interested in sustainable and authentic experiences, thus supporting the growth of the local ecotourism.

## Conclusions

A total of 57 plant taxa and four mushroom taxa were reported and collected. The largest number of plants was reported by Turis (41), followed by Hazaras (37), Khushis (35), and Christians (11). The four studied groups showed some similarities in the plant taxa used: Turis–Khushis share 24 plants, Turis–Hazaras 27, Turis–Christians 8, Khushis–Hazaras 28, Khushis–Christians 9, and Hazaras–Christians 9. Unique or uncommon plants were also used by each of the groups. Turis reported the greatest number of unique plants with 12, while Khushis and Hazaras both reported five, and Christians none. The highest similarity value was observed for Khushis and Hazaras, as the two groups share the same culture and migrated from the same area, namely Afghanistan. Another reason for the similarity between Khushis and Hazaras is that they do normally intermarry. Turis may also intermarry with Khushis and Hazaras, but this is quite rare. Khushis and Hazaras also show some similarity to Turis, but the latter reported the largest number of utilized wild edible plants. This may be due to the fact that Turis depend most on these plants as they live in hilly areas throughout the valley; therefore, Turis collect wild edible plants from mountains and other regions of the valley and sell them in markets. Khushis and Hazaras, on the other hand, mostly depend on the wild edible plants available in markets, as both groups live in the main city of Kurram District and thus have little access to natural areas where wild edible plants are found. Christians show very little similarity to the other groups because they live in the city and depend on jobs in both the public and private sector within the urban perimeter. In order to combat food shortages, local communities in developing nations often rely on wild edible plants for basic food security. Especially for those people who live in the rural and mountainous areas of these countries, wild edible plants may offer a tremendous buffer against food insecurity, which is at the same time culturally appropriate, since foraging is a complex human ecological phenomenon that has been articulated throughout history through a continuous co-evolution of biological and cultural diversities. Applied ethnobotanical (and ethnomycological) knowledge may also help impoverished families who are unable to frequently purchase food from shops and especially in locations where some part of the population is struggling and unable to live a comfortable life. The gathering, processing, cooking, and consuming of wild plants, or more generally, ethnobotanical knowledge, may represent a key to improving local communities’ wellbeing, which needs to be preserved and vividly revitalized through educational platforms.

## Data Availability

All data have already been included in the manuscript.
